# Humoral SARS-CoV-2 Immune Response in COVID-19 Recovered Vaccinated and Unvaccinated Individuals Related to Post-COVID-Syndrome

**DOI:** 10.3390/v15020454

**Published:** 2023-02-06

**Authors:** Catharina Gerhards, Maximilian Kittel, Volker Ast, Peter Bugert, Matthias F. Froelich, Michael Hetjens, Verena Haselmann, Michael Neumaier, Margot Thiaucourt

**Affiliations:** 1 Institute for Clinical Chemistry, Medical Faculty Mannheim of the University of Heidelberg, Theodor Kutzer Ufer 1-3, 68167 Mannheim, Germany; 2 Institute of Transfusion Medicine and Immunology, Heidelberg University, 68167 Mannheim, Germany; 3 Medical Faculty Mannheim, European Center for Angioscience (ECAS), Heidelberg University, 68167 Mannheim, Germany; 4 Department of Radiology and Nuclear Medicine, University Medical Center Mannheim, Heidelberg University, Theodor-Kutzer-Ufer 1-3, 68167 Mannheim, Germany; 5 Department of Biomedical Informatics, Center for Preventive Medicine and Digital Health, Medical Faculty Mannheim, Heidelberg University, 68167 Mannheim, Germany

**Keywords:** antibody dynamics, antibody kinetics, anti-SARS-CoV-2 antibodies, longitudinal assessment, post-COVID syndrome, serological immune response, post-vaccination boosting

## Abstract

Background: The duration of anti-SARS-CoV-2-antibody detectability up to 12 months was examined in individuals after either single convalescence or convalescence and vaccination. Moreover, variables that might influence an anti-RBD/S1 antibody decline and the existence of a post-COVID-syndrome (PCS) were addressed. Methods: Forty-nine SARS-CoV-2-qRT-PCR-confirmed participants completed a 12-month examination of anti-SARS-CoV-2-antibody levels and PCS-associated long-term sequelae. Overall, 324 samples were collected. Cell-free DNA (cfDNA) was isolated and quantified from EDTA-plasma. As cfDNA is released into the bloodstream from dying cells, it might provide information on organ damage in the late recovery of COIVD-19. Therefore, we evaluated cfDNA concentrations as a biomarker for a PCS. In the context of antibody dynamics, a random forest-based logistic regression with antibody decline as the target was performed and internally validated. Results: The mean percentage dynamic related to the maximum measured value was 96 (±38)% for anti-RBD/S1 antibodies and 30 (±26)% for anti-N antibodies. Anti-RBD/S1 antibodies decreased in 37%, whereas anti-SARS-CoV-2-anti-N antibodies decreased in 86% of the subjects. Clinical anti-RBD/S1 antibody decline prediction models, including vascular and other diseases, were cross-validated (highest AUC 0.74). Long-term follow-up revealed no significant reduction in PCS prevalence but an increase in cognitive impairment, with no indication for cfDNA as a marker for a PCS. Conclusion: Long-term anti-RBD/S1-antibody positivity was confirmed, and clinical parameters associated with declining titers were presented. A fulminant decrease in anti-SARS-CoV-2-anti-N antibodies was observed (mean change to maximum value 30 (±26)%). Anti-RBD/S1 antibody titers of SARS-CoV-2 recovered subjects boosted with a vaccine exceeded the maximum values measured after single infection by 235 ± 382-fold, with no influence on preexisting PCS. PCS long-term prevalence was 38.6%, with an increase in cognitive impairment compromising the quality of life. Quantified cfDNA measured in the early post-COVID-19 phase might not be an effective marker for PCS identification.

## 1. Introduction

The coronavirus disease 2019 (COVID-19) pandemic caused by the novel, severe acute respiratory syndrome coronavirus 2 (SARS-CoV-2) was associated with high global mortality and thus calls for adequate concepts to test long-term immunity and identify populations at risk [1,2]. The presentation of COVID-19 is highly variable, with symptoms ranging from inapparent to severe ventilation-requiring pneumonia, but also displays characteristic symptoms such as loss of smell and taste senses (anosmia and ageusia). Further general symptoms are fever, cough, night sweats, loss of appetite, nausea, diarrhea, and muscle pain [3,4]. Beyond these acute symptoms, the occurrence of a post-COVID syndrome (PCS) is a consequence that impacts the health status of COVID-19 convalescents and can affect the quality of life of these patients, despite a recovery from the acute disease [5,6]. A PCS is defined as symptoms that persist 12 weeks after acute COVID-19 or the occurrence of new symptoms associated with previous SARS-CoV-2 infection [7,8]. The appearance of a PCS is a frequent consequence and was detected in this study at a 6-month follow-up in 46.8% of the subjects [9]. Pathognomonic symptoms, such as loss of smell and taste, were most commonly reported. The existence of these long-term effects even 1 year after the detection of SARS-CoV-2 infection are highly relevant for those who have recovered from COVID-19. Furthermore, for a deeper understanding of PCS and humoral long-term immunity, it is essential to consider a correlation between the existence of PCS and SARS-CoV-2-antibody dynamics.

Antibody dynamics in early post-disease stages are already well studied, and an occurrence of seroconversion 7–14 days after a positive qRT-PCR, with a peak at day 30 to 35 post-symptom onset, has been described [10,11,12,13]. In addition, long-term immunity of up to 6 months has been reported in various studies [14,15,16,17,18]. However, verification of long-term immunity beyond 12 months in the clinical context of PCS remains to be verified. The issue of immunity includes both humoral and cellular systems. Considering the implementation of a system that is routinely applicable with high frequency and cost-efficiency, we have focused on the humoral immune response. Since a correlation between the receptor-binding domain (RBD) of the spike (S) 1 subdomain of SARS-CoV-2 and a protective potency was already demonstrated in neutralization tests, these antibodies were used to answer the question of humoral long-term immunity [19,20,21]. In addition, this allows us to compare the results with our 6-month preliminary work consistently. In the context of the current vaccination strategy, the issue of long-term immunity is also related to boosting through vaccination. According to the current German Standing Committee on Vaccination (STIKO) recommendation, convalescents are vaccinated once 6 months after recovery from COVID-19, using one of the currently available vaccines, such as mRNA vaccine NT162b2 (Pfizer-BioNTech, Comirnaty), mRNA vaccine mRNA-1273 (Moderna, Spikevax) or adenovirus-vectored vaccine ChAdOx1 nCoV-19 (AstraZeneca) [22].

A previous study found a reducing effect on PCS through vaccination prior to infection [23,24,25]. Whether a vaccination of COVID-19-recovered subjects influences the further development of a preexisting PCS remains to be investigated and, therefore, will be considered in this study. In this context, we investigated a potential biomarker, cell-free DNA (cfDNA), that is released into the bloodstream from various dying tissues. For other infectious diseases, such as *Staphylococcus aureus* bacteremia or pneumonia, cfDNA concentrations have been identified as a biomarker for differentiating between poor and good outcomes. As a dynamic marker for cell decay, cfDNA could provide information on organ damage in the late recovery phase of COVID-19 [26,27,28,29,30]. It would, therefore, be conceivable that, particularly in the case of persistent lung damage presenting as respiratory symptoms of PCS, increased cfDNA is released into the blood circulation due to fibrotic remodeling of the lung tissue.

Thus, in this work, we present 1-year results of a study assessing longitudinal antibody dynamics in the context of clinical symptoms of 49 COVID-19-recovered subjects infected before vaccination. The aim of this study is (I) to examine the 12-month RBD/S1 and the nucleocapsid (N) protein antibody titer of unvaccinated recovered subjects and of participants vaccinated after infection. The second study aim is (II) to identify parameters that correlate with declining antibody levels after 1 year and thus might be integrated into a predictive model for identifying populations of risk. Moreover, we will investigate the prevalence and symptoms of a PCS 1 year after study inclusion (III). In this context, we will quantify cfDNA in the blood of recovered subjects, to prove its applicability to differentiate between subjects that will suffer from PCS and those without long-term sequelae.

## 2. Materials and Methods

### 2.1. Participant Recruitment and Sample Collection

From April 2020 to October 2021, subjects aged 18 years and older were enrolled in the Immunitor Study at University Medical Center Mannheim, Germany, after recovery from SARS-CoV-2 infection confirmed by qRT-PCR. The Institutional Review Board (2020-556N) approved the study protocol, and the study was conducted in accordance with the Declaration of Helsinki. Research Electronic Data Capture (REDCap), a secure web platform for online data acquisition, was used for the initial standardized survey of medical and personal data at the study entrance, as described in more detail in preliminary work [9]. The assessment of PCS-associated symptoms reported 6 months after study entry was repeated at 12 months after study enrolments.

**Figure 1:**
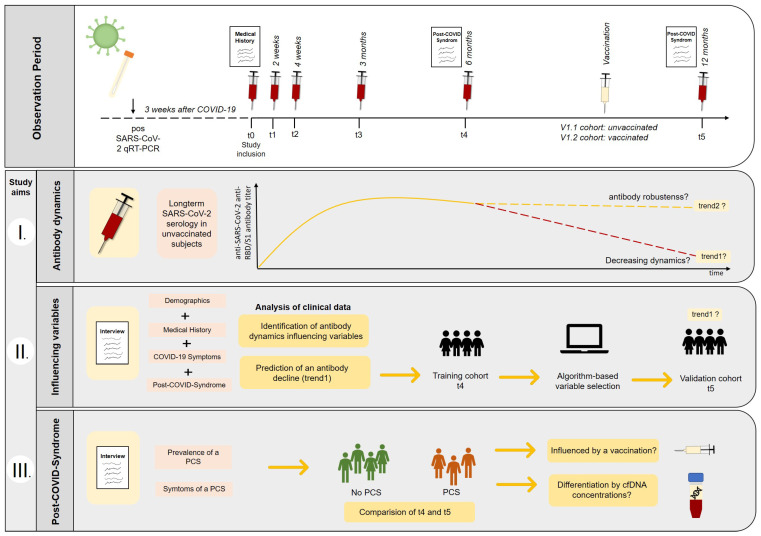
Observation period and study aims. Recovered COVID-19 subjects were enrolled into the study and longitudinal follow-up was performed, including blood sampling and structured interviews. I. Long-term SARS-CoV-2 serology was monitored in unvaccinated subjects to assess declining (trend1) and stable antibody dynamics (trend2). Moreover, the booster effect of vaccination was investigated. II. Clinical data were examined to find variables that could help to identify subjects having a higher risk for declining anti-SARS-CoV-2-antibody titers (trend1). A random forest algorithm was used to identify potential dynamics predicting variables based on the t4-training (6 months) cohort, and potential models were validated using unvaccinated t5 subjects (12 months). III. The prevalence and the symptoms of a post-COVID-syndrome (PCS) were compared between t4 (6 months) and t5 (12 months) appointments. Furthermore, the discriminatory potential of cfDNA concentrations collected in the early post-disease phase (t0) was evaluated, and the impact of vaccination on a preexisting PCS was considered.

For longitudinal surveillance of the humoral SARS-CoV-2 response and further analysis, 7.5 mL serum and lithium–heparin plasma were collected six times (t): study enrollment; post-enrollment 2 weeks; 1 month; 3 months; 6 months; and 12 months (Figure 1). After adequate clotting of serum samples for 1 hour at room temperature, samples were centrifuged at 2000× *g* for 10 min at 18 °C. Plasma was centrifuged identically immediately following sample collection. Serum and plasma were aliquoted and stored at −80 °C until analysis. From t0–t3 SARS-CoV-2, antibodies were measured by use of serum. From t4–t5 anti-SARS-CoV-2, serology was determined using lithium–heparin plasma. Comparison measurements were performed beforehand.

### 2.2. Long-Term Appointments and Definition of Cohorts

**Figure 2:**
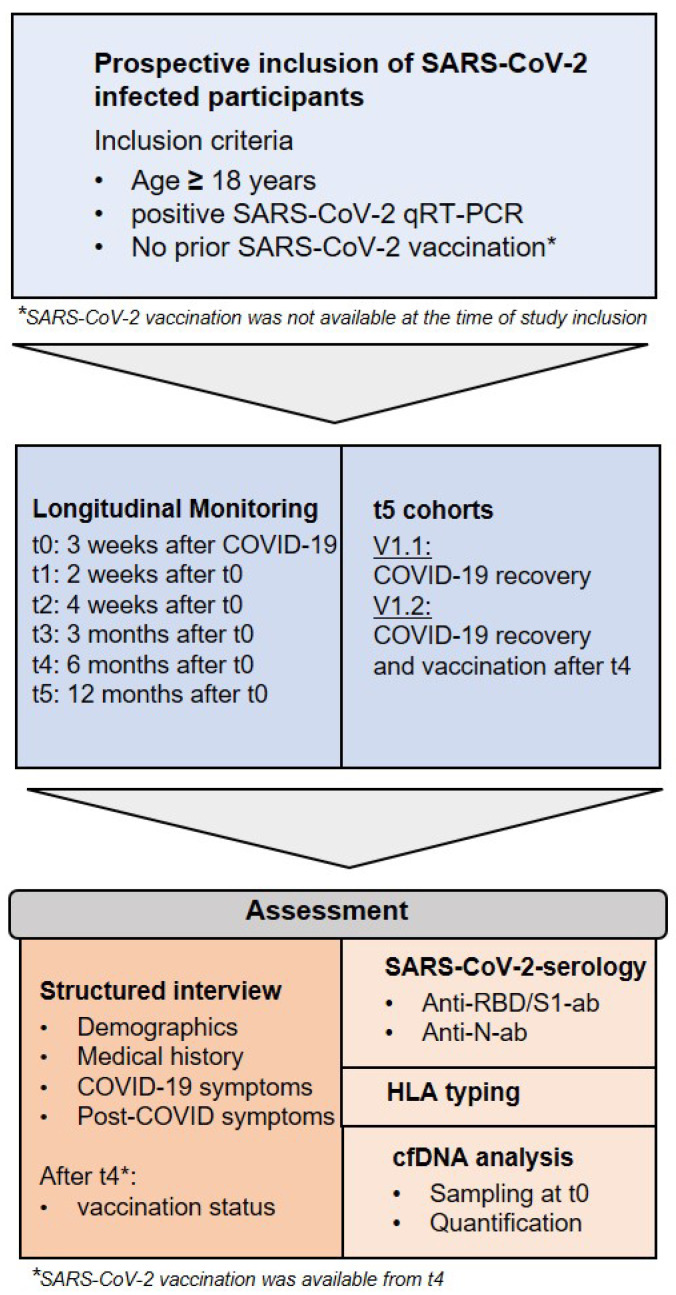
Inclusion criteria and study design.

SARS-CoV-2 infected subjects confirmed by qRT-PCR were included in the study at least 3 weeks after recovery from COVID-19 disease (t0). These subjects were monitored longitudinally for the development of anti-SARS-CoV-2-anti-N- and anti-RBD/S1-antibodies at defined time intervals after study inclusion: t1: 2 weeks; t2: 4 weeks; t3: 3 months; t4: 6 months; t5: 12 months (see Figure 2 and Table 1). The results up to t4 have been published previously. Therefore, the results from t5 (12-month appointment) are presented in this work.

SARS-CoV-2-infected subjects (V1) were longitudinally observed for up to 12 months. Between t4 and t5 appointments, some subjects have been vaccinated. Therefore, two subpopulations were created: V1.1 (unvaccinated at t5) and V1.2 (vaccinated at t5). SARS-CoV-2 serology was measured at each appointment (t0–t5). Moreover, HLA typing and cfDNA analysis were performed at t0. Most common HLA types were examined for an association with SARS-CoV-2-S/RBD1-antibody decline. The cfDNA concentrations were evaluated to differentiate between subjects with and without a PCS.

V1 cohort: From the study baseline, the V1 cohort represented our verum cohort. The verum cohort consisted of qRT-PCR-confirmed recovered COVID-19 subjects. Furthermore, non-SARS-CoV-2-infected control groups were enrolled at t0, which were not monitored longitudinally. “Verum” thus refers to participants infected with COVID-19. Since no vaccines were available at the time of study initiation during the first COVID-19 pandemic wave in April 2020, this represents a cohort demonstrating a disease-related antibody response. The results of t0–t4 have been published in preliminary works and are not discussed in the following, but are presented for comprehension of the study design. Therefore, the data from the t4 (6-months) appointment are used only as a comparison reference to t5 (12 months) and as a training cohort.

V1.1 cohort: Between the 6- (t4) and 12-month (t5) appointments, some subjects had been vaccinated independently of the study design. Therefore, antibody dynamics after disease were assessed only in unvaccinated individuals, the V1.1 cohort.

Trend 1: Insofar as a decrease in SARS-CoV-2-S/RBD1-antibodies was observed in unvaccinated participants, it was described as trend 1. A decrease was defined as a reduction > 10% from the previous value. Influencing clinical variables were examined for the occurrence of trend 1. Potential models were created for predicting an antibody decline (trend 1), described in more detail below.

Trend 2: This represents a stable SARS-CoV-2-S/RBD1-antibody titer with a maximum reduction <10% compared to the previous value. Both trends were studied only in unvaccinated recovered subjects (V1.1.)

V1.2 cohort: Participants that were SARS-CoV-2 vaccinated between their 6-month and 12-month appointments were classified as V1.2 cohort. All V1.2 subjects were infected prior to vaccination.

T5 validation cohort: The training and validation cohorts were used to create clinical models that might predict a reduction in antibody levels and thus help to identify vulnerable patients at higher risk of SARS-CoV-2 reinfection. Only non-vaccinated COVID-19-recovered subjects (12-month appointment, t5) served as a validation cohort to avoid the influence of antibody dynamics due to vaccination. In summary, the V1.1 cohort served as the validation cohort.

T4 training cohort: The training cohort consisted of all 6-month (t4) participants who were not categorized into the validation cohort at t5 to ensure independent training and validation cohorts. All participants, who were later classified as V1.2, served as the training cohort. For the training, the data from t4 (6 months) were used to avoid any influence by a booster effect.

### 2.3. Anti-SARS-CoV-2 Antibody Detection and HLA Typing

Anti-SARS-CoV-2 anti-N and anti-RBD/S1 antibody detection was performed with the same platform, as described in preliminary work [9]. Lithium–heparin samples were centrifuged at 2000× *g* for 10 min at 4 °C; plasma was aliquoted and stored at −80 °C until use. A qualitative, CE- and FDA-approved electrochemiluminescence immunoassay (ECLIA) determining anti-SARS-CoV-2 anti-N Pan-Ig (including IgG and IgM) was considered positive at a cut-off index (COI) ≥ 1.0. The quantitative Elecsys^®^ Anti-SARS-CoV-2 S (Roche, Germany) test, likewise CE-marked and FDA-approved, was used to analyse anti-SARS-CoV-2 anti-RBD/S1 total Ig and results were classified as reactive from a titer of 0.8 U/mL. The analyses were conducted in accordance with the manufacturer’s instructions and after internal verification in line with ISO 15189 in an accredited laboratory. In cooperation with the Institute of Transfusion Medicine and Immunology, Heidelberg University, Medical Faculty Mannheim, German Red Cross Blood Service Baden-Württemberg—Hessen, Mannheim, Germany, HLA typing was performed according to the standard protocol.

### 2.4. Analysis of cfDNA

At t0, study inclusion, 9 mL ethylene diamine tetraacetic acid (EDTA, Sarstedt, Germany) blood was collected for cfDNA isolation, and plasma was separated within 4 h of blood sampling. For plasma separation, the tubes were centrifuged at 1600× *g* for 10 min at 20 °C; the supernatant was transferred to a further 15 mL tube and then centrifuged at 3000× *g* for 10 min. Hemolytic samples identified by optical control were excluded. Plasma was stored at −80 °C until further analysis. We isolated cfDNA using the Plasma cfDNA Extraction Kit (Vienna Lab, Vienna, Austria) and QIAmp Circulating Nucleic Acid Kit (Qiagen, Hilden, Germany), depending on the availability. The isolation was performed according to the manufacturer’s instructions without modifications, utilizing 2 mL plasma volume from each subject. Comparison measurements of the isolation methods were performed. The cfDNA was quantified using the Qubit Fluorometer (ThermoFisher, Life Technologies Corporation, Madison, WI, USA). The concentration was recalculated in relation to the input volume and reported as ng per mL plasma cfDNA.

### 2.5. General Data Analysis

Clinical data acquisition was systematically performed via the REDCap platform. Statistical analyses were performed in R (version 4.1.1) and in Microsoft Excel Version 2019 (Microsoft, Redmond, WA, USA). For all statistical analyses, *p*-values < 0.05 were considered statistically significant. Graphs were plotted using GraphPad 7.0, R (version 4.1.1) or Microsoft PowerPoint Version 2019 (Microsoft, Redmond, WA, USA).

### 2.6. Performance of Feature Selection

For establishing a clinical prediction model of declining Anti-SARS-CoV-2 anti-RBD/S1 dynamics, we used a random forest approach, assessing the importance of potential predictor variables by comparison with so-called shadow variables. These shadow variables are generated for each real variable by permutation and a statistical test is performed, comparing the importance of the real variables and the maximum importance of all shadow variables. If the real variable is significantly more important than the shadow variable, the algorithm assigns high importance to the feature [31,32]. The training set included all t4 subjects who were not categorized into subpopulation V1.1, unvaccinated recovered subjects. The following parameters were included in this training dataset: COVID-19 symptomatic (fever, night sweat, diarrhea, cough with and without sputum, shortness of breath, muscle pain, nausea, loss of smell or taste, other symptoms), treatment type (ambulatory, inpatient, ICU), preexisting medical history (immune, autoimmune, vascular, pulmonary, gynecological, cancer and other diseases), medications, blood group, gender, age, BMI, smoking status, the existence of PCS, and symptom differentiation of PCS (olfactory, gustatory or respiratory impairment, fatigue, exhaustion, hair loss, cognitive symptoms, or depression). Kidney and liver diseases were likewise inquired during the initial anamnesis. Still, since none of the training cohort subjects reported aforementioned disease conditions of these types, an inclusion in the feature selection was not feasible. As a validation dataset, the results of the t5 appointment of the V1.1 cohort were used, containing the identical features listed above. For statistical computing, the R (https://www.r-project.org (accessed on 5 January 2022)) version 4.1.1 software was utilized.

## 3. Results

### 3.1. Demographics and Overall Antibody Trends

Forty-nine of the 61 subjects enrolled at the study baseline participated at the annual appointment, t5. Between 18 and 495 days post-positive qRT-PCR-result, 325 serum and lithium–heparin samples were collected. Demographic data of the 1-year cohort t5 compared to t4 are presented in Table 2. Furthermore, the characteristics of V1.1 (unvaccinated t5) and V1.2 (vaccinated t5) are illustrated in Table 3. The median age of participants undergoing 12-month follow-up was 53.1 years (ranging from 21–78 years); 65.3% of the participants were female (f/m 33/17); and mean body mass index (BMI) was 25.8 (BMI range 19–36). Among t5 subjects, 6.2% (4/49) were smokers and 93.8% were non-smokers when interviewed. Concerning acute COVID-19 symptoms, olfactory (35/48; 72.9%) and gustatory dysfunctions (38/48; 79.2%) (both: 39/48; 81.2%) and fever (25/48; 52.1%) were most frequently reported. In the acute phase of COVID-19, 12.5% of the t5 cohort required hospitalization; one-third of them needed intensive care; whereas most subjects did not require inpatient treatment. Concerning the existence of a PCS, 46.9% of the subjects presented long-term sequelae after 6 months, while 38.6% suffered from a PCS in the course of 1 year.

**Figure 3:**
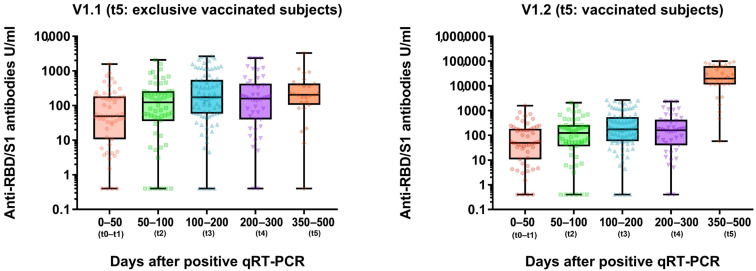
Longitudinal anti-SARS-CoV-2-anti-RBD/S1 dynamic (U/mL) in recovered COVID-19 subjects. V1.1 cohort of t5 appointment includes only unvaccinated subjects (**left** figure). Vaccinated subjects were included in the V1.2 cohort at t5 (**right** figure).

At the half-year appointment (t4), anti-SARS-CoV-2 anti-N antibodies were detected in 46/49 and anti-SARS-CoV-2 anti-RBD/S1 antibodies were assessed in 47/49. At t5, anti-SARS-CoV-2 anti-RBD/S1 antibodies were detected in 48/49 and anti-N antibodies in 46/49 subjects (Figure 3). All subjects’ characteristics and antibody trends are compared in Table 2 and Table 4. Individual antibody dynamics are presented in Figure 3. The volunteer with significant, namely a 20-fold anti-N-antibody, increase suffered a second COVID-19 disease between t4 and t5. Moreover, a 138-fold increase in the anti-SARS-CoV-2 anti-RBD/S1 antibodies could be attributed to the reinfection, as no vaccination was performed before the blood collection. No further qRT-PCR-confirmed reinfection occurred among the other participants and no indicative symptoms were reported.

### 3.2. Anti-RBD/S1 Antibody Dynamics in Unvaccinated (V1.1) and Vaccinated Population (V1.2)

**Figure 4:**
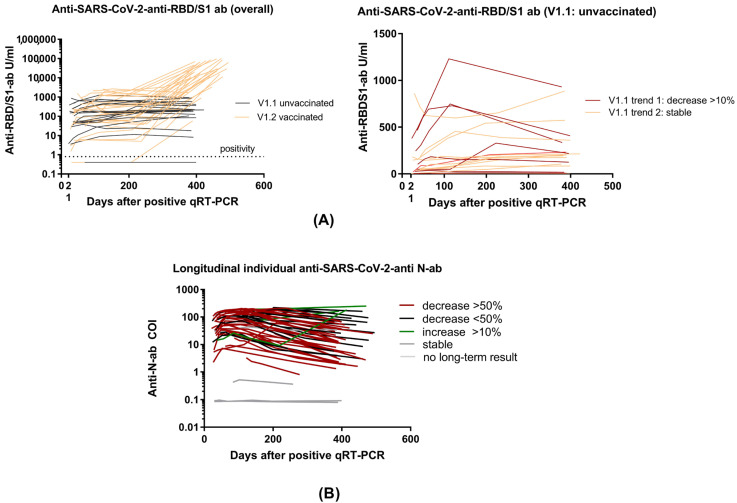
Individual longitudinal Anti-SARS-CoV-2-antibody dynamics. (**A**) Long-term individual anti-SARS-CoV-2-RBD/S1 antibodies in overall cohort differentiated between vaccinated and unvaccinated subjects (left figure). V1.1 unvaccinated divided into trend 1 (decreasing antibodies) and trend 2 (increasing antibodies). (**B**) Long-term individual anti-SARS-CoV-2-N-antibodies (COI). A decrease of more than 50% is illustrated by the color red, a decrease < 50% by the color black, and an increase of >10% by the color green. Stable levels and subjects without long-term results are presented in gray.

12-month antibody dynamics were considered separately in the vaccinated (V1.2, *n* = 29) and unvaccinated (V1.1., *n* = 18) subpopulations (Figure 3). The unvaccinated population was furthermore divided into group V1.1._trend1 with decreasing anti-RBD/S1 antibody dynamic (>10% from previous value, *n* = 7) and V1.1_trend2 with stable to increasing anti-RBD/S1 antibody levels (*n* = 12) to identify potential parameters for anti-RBD/S1 antibody decreased (Figure 4A). One subject in the V1.1._trend2 group had a negative titer during the entire study and was therefore not included in the following comparison. Mean anti-SARS-CoV-2 anti-RBD/S1 antibodies showed no significant difference, with a median value of 218.8 U/mL for V1.1._trend2 and 203.0 U/mL for V1.1. (*p* = 0.770). Considering the mean changes compared to prior values, we observed a significant deviation between the two collectives, with a mean value of 112.0 ± 32.0% in V1.1_trend2 and a value of 68.5 ± 9.9% in V1.1_trend1 (*p* = 0.001).

Therefore, various factors were investigated concerning their influence on the anti-SARS-CoV-2 anti-RBD/S1 antibody declining dynamics and are discussed here. Comparing the V1.1 subpopulations, there was no significant difference in gender (Fisher’s exact test: *p* = 1.0) and age (*p* = 0.101). Only a slight tendency of a lower age in V1.1_trend1, with a mean value of 39.7 ± 11.9 years and a mean age of 50.5 ± 12.1 years in V1.1_trend2, was observed. Since a positive correlation between the level of RBD/S1 antibody titer and age was identified in our previous work [9], we further compared the level of antibodies at t4 to assess whether higher baseline levels were associated with an antibody decline. A significant difference in anti-RBD/S1 antibody levels between the two subpopulations could not be detected at the t4 appointment (*p* = 0.222). Compared to the maximum anti-RBD/S1 antibody titer (*p* = 0.176), there was also no significant difference, thus we deduced that a higher baseline titer may not be the primary cause of an antibody decline. Further factors correlating with the level of antibodies at t4, namely BMI and severity, were also examined. Regarding the BMI, there was a clear tendency for a higher BMI in the V1.1_trend1, with a mean of 29.9 ± 5.6 (*p* = 0.074). However, no effect was observed for the severity of COVID-19 (*p* = 0.370). As all subjects in the V1.1 collective were non-smokers, the influence of smoking status could not be investigated. In addition, HLA typing was performed to examine the impact on the classification into declining (V1.1_trend1) or stable trends (V1.1._trend2). For this purpose, the alleles most frequently represented in our collective were selected. This involved HLA A*01, HLA A*02, HLA A*24, HLA B*44, HLA C*07, HLA C*04, HLA DR*13, HLA DQ*03, HLA DQ*05, HLA DQ*06 and HLA DP*04. Overall, no significant correlation between the occurrence of an anti-RBD/S1 antibody decrease and one of the investigated HLA types was revealed (Fisher’s exact test: all *p*-values > 0.1).

The vaccinated (V1.2) cohort comprised 29/49 subjects, all of whom had been vaccinated before t5 (12 months) (15 received one dose of vaccine and 14 received two doses). Twenty-two of the subjects were vaccinated purely with mRNA vaccine NT162b2 (Pfizer-BioNTech, Comirnaty), two with mRNA vaccine mRNA-1273 (Moderna, Spikevax), and five with adenovirus-vectored vaccine ChAdOx1 nCoV-19 (AstraZeneca), of which one was vaccinated in heterogeneous combination with an mRNA vaccine. The mean anti-SARS-CoV-2 anti-RBD/S1 antibody titer of the V1.2 cohort was 34,967 ± 31,060 U/mL. There was a mean 235 ± 382-fold antibody increase after vaccination compared to maximum titer after convalescence (*p* < 0.0001; *n* = 29, longitudinal S-antibody dynamics see Figure 4).

### 3.3. Clinical Prediction of Anti-RBD/S1 Antibody Decrease

**Figure 5:**
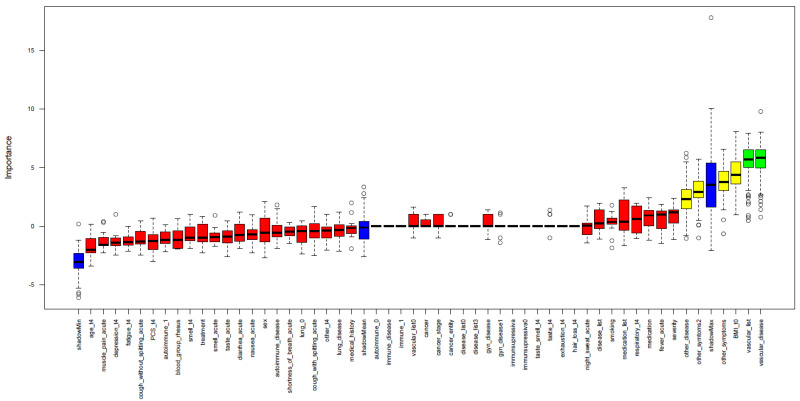
Importance of clinical variables for a decline in S-abs. A random forest algorithm was applied to study clinical variables’ importance in predicting anti-SARS-CoV-2-anti-RBD/S1-ab decline. High importance is illustrated by green; medium importance by yellow; and low importance by red. In addition, the shadow variables’ minimum, mean and maximum importance is shown in blue.

As a singular significant demographic parameter was not identified to predict decreasing anti-SARS-CoV-2 anti-RBD/S1 antibody dynamics, we included additional clinical features, such as medical history and acute symptoms of COVID-19. Thereby, we intended to establish a prediction model (training set t4, *n* = 30), even though no significant differences in the level of anti-SARS-CoV-2 anti-RBD/S1 antibody have been observed in V1.1. trend1 and V1.1. trend2 so far. The exact parameters and use of the feature selection model were described in more detail in the section methods”. “Vascular disease” with further differentiation of the type, as well as “other symptoms” (such as headache and joint pain, fatigue and loss of appetite in the acute phase of the disease) and the quantity of these, were selected as variables with highest importance. The parameters “BMI at t0” and “other preexisting diseases” were assigned medium importance (Figure 5). The prediction was tested for all high and medium importance variables and possible combinations of those. ROC analysis identified the following models as most appropriate: “other disease” (AUC 0.74), “vascular disease and other diseases” (AUC 0.70), and “other disease and other symptoms” (AUC 0.69). The prediction of decreasing antibody dynamics in the validation set (*n* = 18) was performed and revealed the following results: model “other disease” and model “other disease and other symptoms”: 4/7 correctly predicted and one false positive, followed by model “vascular disease and other diseases” and model “BMI and other diseases”: 3/7 correctly identified and one false positive. It should be noted that there is a negative correlation between the incidence of vascular disease and a falling antibody trend.

### 3.4. Identification of Risk Factors or Suitable Biomarker for PCS Occurring

**Figure 6:**
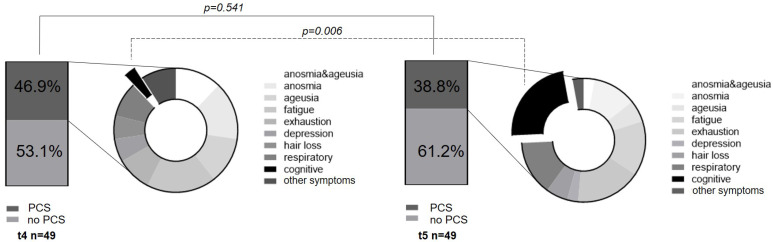
Post-COVID Syndrome dynamics. The existence of PCS at the 6-month and the 12-month appointments is compared. In addition, the distribution of the individual symptoms, and especially the increase in cognitive symptoms, is shown in the pie chart.

In terms of PCS development, in the non-vaccinated V1.1 cohort, 7/18 continued to show COVID-19 long-term sequelae at t5 (t4: 9/18). Concerning the symptom combination of loss of taste and smell, two subjects reported regression (t4: 3/18; t5: 1/18), while the singular expression of anosmia (2/18) and ageusia (1/18) remained constant. Interestingly, there was an increase in cognitive impairments, such as loss of concentration and attention deficits (t5: 3/18). The increase in cognitive symptoms is particularly evident when considering the overall t5 cohort, where 8/49 subjects reported cognitive impairments, thus representing the most common long-term symptom at 1-year follow-up. In addition, the symptoms of fatigue (5/49) and exhaustion (6/49) remained prominent (see Figure 6 and Table 5). Regarding the influence on antibody tendencies in the V1.1 cohort, the existence of a PCS was not identified as a relevant parameter. Within the V1.1_trend2 cohort, 5/11 suffered from a PCS, and in the V1.1.trend1 cohort, 2/7 reported long-term effects (Fisher’s exact test: *p* = 0.637). In the vaccinated (V1.2) cohort, 14 subjects reported long-term sequelae at the 6-month appointment (t4) and 11 subjects still presented long-term effects at the 1-year follow-up. (t5) Thus, there was no significant change in the existence of PCS after vaccination (*p* = 0.596).

Moreover, we collected blood to isolate cfDNA from 23 subjects at the first appointment of the study. The median concentration in the cohort without PCS was 0.13 ng/µL vs in samples with PCS 0.16 mg/µL. Recalculated regarding plasma input, the median concentration was 1.94 ng/mL [IQR: 1.43, 3.53] for subjects with long-term effects and 1.74 ng/mL [IQR: 1.48, 2.86] for subjects without long-term effects. Thus, no significant difference could be observed (*p* = 0.951).

## 4. Discussion

Long-term anti-SARS-CoV-2-antibody-response and the dynamics of PCS are of current interest and have a major impact on large numbers of COVID-19-recovered individuals [33]. In particular, the question of humoral immunity remains essential, even after the global vaccination strategy has been implemented, as seasonal trends towards increasing incidences in colder months are still emerging [13,34]. Moreover, in addition to the acute threat of reinfection, long-term consequences are a severely limiting factor for the quality of life of COVID-19 survivors [33]. Therefore, we addressed antibody dynamics associated with clinical determinants and the development of a PCS in a 1-year follow-up of 49 COVID-19-recovered subjects. These subjects were categorized into two subpopulations: vaccinated and unvaccinated individuals.

First, we address the development of long-term SARS-CoV-2 serology of subjects enrolled during the first pandemic wave. We discuss the 12-month dynamics based on anti-SARS-CoV-2-N-ab and anti-SARS-CoV-2-S/RBD1-ab. In addition to a purely disease-specific serological immune response, we have observed changes due to a vaccination. Previously, we reported overall stable RBD/S1-antibody titers accompanied by already declining N-antibody levels after 6 months of study inclusion [9]. At the 12-month appointment, an even higher decrease in N-antibodies was observed, as more than 85% of the subjects demonstrated a reduction of more than 10% (Figure 3B). Some previous studies have revealed a predominant and more rapid reduction in N-antibodies, as confirmed by our findings [9,35]. One subject with a significant N-antibody increase suffered a reinfection between t4 and t5. Regarding humoral protection, N-seronegativity has been discussed as a risk factor for reinfection [36,37]. In this context, Ali et al. found that 25/87 N-antibody-negative subjects suffered reinfection. Considering N-antibody-positive subjects, only 1/742 was asymptomatically reinfected. This reinfected N-antibody-seropositive subject presented a low N-antibody IgG level of 5.87 s/ca after the first recovery (≥1.1 was evaluated as positive) [36]. Interestingly, our study’s reinfected subject also showed a lower mean N-antibody titer of 16.2 ± 5.5 COI after initial recovery.

However, regarding humoral SARS-CoV-2 immunity, it has been demonstrated that anti-RBD/S1-antibodies correlate significantly with neutralizing capacity in neutralization assays and thus can be utilized as an inexpensive and easier-to-implement assay in routine care [38]. Therefore, in this study, we investigated the evolution of anti-RBD/S1-antibodies in various subpopulations (Figure 3A). In the context of studies with data beyond 1 year after recovered infection, Glöckner et al. showed that 78% of the subjects were S-antibody seropositive at 12-month follow-up [38]. Our data confirm long-lasting anti-RBD/S1-antibodies beyond 1 year, as median percentage antibody dynamic related to the maximum measured value was 101%. Moreover, at 12-month follow-up, only one subject had a reduction of more than 50% compared to the previous value (anti-RBD/S1-antibody half-life). This high seropositivity raises optimism, also concerning the prospect of long-term humoral immunity after vaccination, as similar patterns of decline are to be expected.

Furthermore, in the context of boosting COVID-19-recovered subjects, vaccinated convalescents showed a mean 235 ± 382-fold antibody increase after vaccination compared to maximum titer after recovery (*p* < 0.001), in line with previous findings [38]. This antibody increase illuminates the expected enormous booster effect of a vaccination based on a cohort of subjects vaccinated after COVID-19 recovery (V1.2 cohort). Thus, the differences in anti-RBD/S1-antibodies between V1.1 and V1.2 can be explained by vaccination. Moreover, in our unvaccinated, recovered COVID-19 cohort (V1.1), we observed an overall stable anti-SARS-CoV-2-S/RBD1-ab trend, as only one participant’s antibody titer decreased more than half of the previous antibody level (<50% was defined as half-life). This marginal occurrence of a significant decrease indicates the robustness of the anti-SARS-CoV-2-S/RBD1-ab over a long-term period of at least 12 months (study aim I).

Although the robustness of anti-SARS-CoV-2-S/RBD1-ab was found, we wanted to identify clinical variables that might explain a relative drop >10% in a subcohort of subjects (V1.1 trend 1). We intended to identify parameters that correlate with declining antibody levels after 1 year and thus can be integrated into a logistic model for identifying a population at risk (study aim II). It should be noted that there was no significant difference between the mean anti-RBD/S1-antibody levels in our two unvaccinated cohorts. Hence, no evidence of a high vulnerability in our unvaccinated declining (V1.1_trend1) cohort could be inferred from the antibody levels. However, decreasing trends were observed, providing a validation dataset for the identification of clinical factors which affect antibody dynamics. Considering demographic aspects, neither gender (*p* = 1.0) nor age could be identified as a significant parameter influencing decreasing anti-RBD/S1-antibody titers, although a slight tendency towards lower age was observed to be associated with a higher antibody decrease (*p* = 0.101). These results are supported by Zeng et al., who reported comparable seroprevalence rates in male and female subjects after 1 year, with younger subjects more commonly showing low antibody titers [39].

Several HLA types, such as HLA DRB1*01:01 and HLA B*35:01, have been described to be associated with severe courses in the acute phase of the disease [40,41]. Due to the impact on COVID-19 progression, we investigated HLA genotypes in the unvaccinated (V1.1) cohort with declining antibodies for associations with antibody tendencies. Neither the diverse HLA types (HLA A*01, HLA A*02, HLA A*24, HLA B*44, HLA C*07, HLA C*04, HLA DR*13, HLA DQ*03, HLA DQ*05, HLA DQ*06 and HLA DP*04, all *p* values > 0.1) of the participants nor the severity of the acute illness (*p* = 0.370) were identified as significant parameters in the differentiation between decreasing (V1.1_trend1) and stable (V1.1_trend2) dynamics. In addition, various studies investigated the influence of BMI on anti-SARS CoV-2 seroprevalence [9]. Thus, we have further examined BMI as a trend1 influence in the V1.1 cohort and have identified a slight trend towards higher BMI in declining anti-RBD/S1.

Overall, examining clinical variables to explain the existence of subjects with an anti-SARS-CoV-2-S/RBD1-ab decrease of >10%, only younger age and higher BMI tendencies were revealed, as underlined by previous studies. Therefore, we used a random forest algorithm to identify combinations of clinical variables that might explain the differences. Our clinical prediction model showed that the variables “other disease” (AUC 0.74) and “vascular disease and other diseases” (AUC 0.70) led the closest approximation to the actual subject distribution of falling antibodies. However, the overall predictive power was rather limited. Moreover, the variable “other diseases” included all other illnesses that were not explicitly assessed in the questionnaire. Thus, this variable is not very specific and does not allow direct prediction or association. The same limitation applies to “other symptoms”. However, cardiovascular disease, highly prevalent in our society, which showed passable prediction together with the association of “other diseases”, was not positively correlated with a decrease in antibody levels. This is encouraging, as it might indicate that a reduction in anti-SARS-CoV-2 anti-RBD/S1-antibodies is not promoted, but needs to be verified in follow-up studies with a higher number of subjects.

This leads to some limitations of the study that need to be addressed. One important aspect to discuss is the relatively small size of the dataset due to the high prevalence of vaccination. As we did not want to influence the risk of infection through our study participation, the subjects had already been vaccinated independently of our study protocol, leading to a pure infection-based long-term antibody response tested in a smaller cohort (V1.1). Therefore, this approach needs to be extended and externally tested in larger cohorts to implement a clinical model for identifying risk populations. In terms of the symptom “loss of concentration” and “attention deficits”, it could be suggested that there is an influence from the media, which have described an increase in cognitive symptoms. Still, we have to emphasize that the surveys were conducted before July 2021, before those findings had been published. In addition, it should be noted that quantification via tests of memory and concentration could have provided more objective evidence of these symptoms. As such trends were not known until the study was finished, those surveys were not conducted. However, the influence of a suggestive question can be ruled out, since the information regarding cognitive symptoms was collected via an open question.

This segues into the third aim of the study, as we investigated the dynamics of a PCS 12 months after study inclusion. At the t5 appointment, 38.6% of overall subjects suffered from this syndrome, showing a slight non-significant decreasing tendency compared to t4 (t4: PCS 46.9, *p* = 0.541). Regarding the pathognomonic loss of smell and taste, there was a slight, but not significant, tendency of reconstitution of sensory perception (*p* = 0.356). Likewise, in either anosmia or ageusia, the persistence of symptoms beyond 1 year after the disease occurred (anosmia: *p* = 1.0; ageusia: *p* = 0.673, Table 4). However, the sensory-affected subjects stated that there was a subjective improvement compared to the acute phase of the disease. Regarding quality, the acute symptoms were described mainly as hyposmia/dyssomnia or hypogeusia/dysgeusia. Riestra-Ayora et al. also described such long-term changes in a 6-month course. Here, 11% of the subjects showed persistent sensory symptoms compared to the acute disease, which presented as olfactory and gustatory dysfunction [42]. We found a comparably significant decrease (4/35) in anosmia between acute COVID-19 and t5 with persistence in 11.4% of subjects (*p* < 0.001 **). This supports and even extends previous results over a longer period [33,42]. Beyond these characteristic symptoms of the acute phase, the long-term monitoring revealed an evident change from the initial sensory symptoms to cognitive manifestations, such as concentration and attention deficits. Interestingly, these complaints increased significantly at t5 (t5: 8/19, t4: 1/23, *p* = 0.006) and, together with fatigue (5/19) and exhaustion (6/19), were reported as particularly affecting the quality of life. We compared vaccinated and unvaccinated subjects regarding the dynamic of a PCS, and no significant difference in the existence of a PCS at t4 and t5 could be observed. This indicates that vaccination after COVID-19 recovery might not influence the evolution of a preexisting PCS.

Regarding the existence of a PCS, we further examined cfDNA, a dynamic marker of cell decay, for its suitability to distinguish between subjects that might suffer from a PCS and participants not affected by long-term sequelae. In previous studies, higher concentrations of cfDNA were observed in infectious diseases and identified in *Staphylococcus aureus* bacteremia and acute COVID-19 infection as a biomarker of a fulminant course [27,28,30]. Therefore, we collected blood to isolate cfDNA from 23 subjects at the first appointment of the study. We assessed the occurrence of PCS at t4 and t5 appointments and were thus able to compare the cfDNA concentrations of both cohorts. No significant difference in cfDNA concentrations between subjects with and without PCS was evident (*p* = 0.601). Hugon et al. observed that the described cognitive impairment, so-called “brain fog”, is associated with hypometabolism measurable by abnormal FDG PET findings in the cingulate cortex [43]. Since cfDNA is a marker of cell death and not of cerebral hypoperfusion, this may explain why cfDNA might not be a suitable biomarker of a PCS, especially regarding cognitive complaints.

## 5. Conclusions

In conclusion, we observed overall stable anti-RBD/S1-antibody levels in unvaccinated individuals in long-term follow-up, as median percentage antibody level related to the maximum measured value was 101%. In addition, only one participant’s antibody titer decreased more than half of his previous antibody level. Moreover, we demonstrated an enormous booster effect of vaccination on anti-RBD/S1 antibody levels in individuals who had recovered from COVID-19 before they were vaccinated. Significantly decreasing trends were seen with regard to anti-N antibodies, as 85% of the subjects had a decline, and 55% even showed a drop of more than half of the previous value. The investigated HLA types had no influence on our cohort’s longitudinal antibody level tendency. Although our prediction model was based on clinical patient information, no concrete pre-existing condition or COVID-19 symptom permitted adequate prediction of antibody level decrease. According to current research, vaccination may reduce the risk of PCS. However, our results suggest that vaccination after breakthrough infection provides only partial protection against PCS, as there was no statistical difference in the frequency of PCS between the vaccinated and unvaccinated cohort. After 1 year, a persistent PCS prevalence of 38.6% with an increase in cognitive impairment was revealed, compromising the quality of life of affected subjects. The presence of cfDNA in ETDA-plasma as an indicator for acute severe tissue damage was not significantly higher in PCS patients than without PCS and hence seemed inadequate as an effective early biomarker for the development of PCS.

## Figures and Tables

**Table 1:** Cohorts and trends.

Cohort	Description
t0	Study inclusion (at least 3 weeks after COVID-19, no prior SARS-CoV-2 vaccination)
t1	2 weeks after t0
t2	4 weeks after t0
t3	3 months after t0
t4	6 months after t0
t5	12 months after t0 (some subjects were vaccinated before t5)
V1	Recovered SARS-CoV-2-infected unvaccinated participants from t0–t4
V1.1	Recovered SARS-CoV-2-infected unvaccinated participants at t5
V1.2	Recovered SARS-CoV-2-infected and vaccinated participants at t5
Training	Data from V1.2. at t4
Validation	Identical with V1.1
Trend 1	Declining anti-S/RBD1-ab in unvaccinated subjects (≥10% to previous value)
Trend 2	Stable anti-S/RBD1-ab in unvaccinated subjects

**Table 2:** Comparison of COVID-19 recovered subjects 6 and 12 months after study inclusion (all V1 subjects).

	T4 (*n* = 49)	t5 (*n* = 49)	*p*-Value
Age (median [IQR])	51.00 [32.50, 58.25]	53.12 [35.48, 60.02]	0.462
Gender f/m (%)	31/18 (63.3/36.7)	33/17 (65.3/34.7)	1
Smoking (%)	3 (8.3)	4 (6.2)	1
BMI t0 (mean (SD))	25.50 (4.61)	25.82 (4.77)	0.74
**Treatment (%)**			
Ambulant	42 (87.5)	42 (87.5)	
Hospitalization: normal	4 (8.3)	4 (8.3)	
Hospitalization: ICU	2 (4.2)	2 (4.2)	
COVID-19 symptoms			
Fever (%)	25 (52.1)	25 (52.1)	1
Anosmia COVID-19 (%)	37 (77.1)	35 (72.9)	0.814
Ageusia COVID-19 (%)	39 (81.2)	38 (79.2)	1
Anosmia and Ageusia (%)	40 (83.3)	39 (81.2)	1
Days after qRT-PCR (mean (SD))	243.81 (44.49)	414.18 (35.03)	<0.001
Vaccination before the appointment (%)	0 (0.0)	30 (61.2)	<0.001
**Preexisting medical history**			
Autoimmune (%)	8 (16.7)	8 (16.7)	1
Immune (%)	1 (2.1)	1 (2.1)	1
Vascular (%)	9 (18.8)	12 (25.0)	0.622
Diabetes (%)	1 (2.1)	1 (2.1)	1
Cancer (%)	1 (2.1)	1 (2.1)	1
Lung (%)	4 (12.5)	8 (16.7)	0.773
Other diseases (%)	14 (29.2)	13 (27.1)	1
Medication (%)	24 (50.0)	27 (56.2)	0.683

**Table 3:** Comparison of recovered unvaccinated (V1.1) and recovered vaccinated (V1.2) subjects at t5 (12-month appointment).

	Recovered, Unvaccinated V1.1 (*n* = 19)	Recovered, Vaccinated V1.2 (*n* = 29)	*p*-Value
Anti-RBD/S1 antibodies			
Titer U/mL (median [IQR])	204.30 [115.30, 357.95]	20,331.00 [11,497.00, 62,414.00]	<0.001
Comparison previous t % (median [IQR])	103.00 [73.48, 109.69]	13,574.76 [5798.89, 29,051.89]	<0.001
Dynamics (%)			<0.001
Stable	7 (36.8)	0 (0.0)	
Increase	5 (26.3)	28 (96.6)	
Decrease	7 (36.8)	1 (3.4)	
PCS t4 (%)	9 (47.4)	14 (48.3)	1
PCS t5 (%)	8 (42.1)	11 (37.9)	1

**Table 4:** Comparison of SARS-CoV-2 ab of COVID-19 recovered subjects after 6 and 12 months (all subjects).

	6 Months (t4) (*n* = 49)	12 Months (t5) (*n* = 49)	*p*-Value
Anti-SARS-CoV-2-anti-RBD/S1 antibodies			
Titer U/mL (median [IQR])	172.10 [48.99, 506.20]	3519.00 [218.80, 29,354.00]	<0.001
Comparison previous t % (median [IQR])	106.85 [96.16, 134.37]	4077.99 [105.10, 14,520.00]	<0.001
*Dynamics (%)*			0.020
Stable	15 (30.6)	7 (14.3)	
Increase	20 (40.8)	34 (69.4)	
Decrease	14 (28.6)	8 (16.3)	
*Dynamics unvaccinated cohort V1.1 (%)*			0.558
Stable	15 (30.6)	7 (36.8)	
Increase	20 (40.8)	5 (26.3)	
Decrease	14 (28.6)	7 (36.8)	
**Anti-SARS-CoV-2 anti-N-antibodies**			
Titer COI (median [IQR])	32.82 [8.33, 94.69]	13.78 [4.01, 51.89]	0.109
*Dynamics (%)*			0.735
Stable [negatives]	5 [1] (10.2)	4 [3] (8.2)	
Increase	5 (10.2)	3 (6.1)	
Decrease	39 (79.6)	42 (85.7)	

**Table 5:** Comparison of PCS prevalence and symptoms of recovered subjects 6 and 12 months after study inclusion (all subjects, V1).

	t4 (*n* = 49)	t5 (*n* = 49)	*p*-Value
PCS (%)	23 (46.9)	19 (38.6)	0.541
*PCS Dynamics*			
Regression	Na	9 (18.4)	
Persistence	Na	14 (28.6)	
Initial manifestation	Na	5 (10.2)	
Perpetual asymptomatic	Na	21 (42.9)	
*PCS Characteristics*			
Anosmia and ageusia (%)	4 (17.4)	1 (5.3)	0.356
Anosmia	5 (21.7)	4 (21.1)	1
Ageusia	4 (17.4)	2 (10.5)	0.673
Fatigue	6 (26.1)	5 (23.3)	1
Exhaustion	3 (13.0)	6 (31.6)	0.257
Depression	2 (8.7)	1 (5.3)	0.582
Loss of hair	2 (8.7)	2 (10.5)	1
Respiratory symptoms	3 (13.0)	4 (21.1)	0.682
Cognitive impairments	1 (4.3)	8 (42.1)	0.006
Other symptoms	3 (13.0)	1 (5.3)	0.614

## Data Availability

The data presented in this study are available on request from the corresponding author.
